# Prospects of engineered bacteria-assisted CAR T Cell therapy in gastrointestinal cancers

**DOI:** 10.3389/or.2025.1581856

**Published:** 2025-04-14

**Authors:** Qingqing Zhang, Xiao Song, Junhong Liu, Xuejiao Zhou

**Affiliations:** ^1^ Reproductive Medicine, Weifang People’s Hospital, Weifang, Shandong, China; ^2^ Department of Gastroenterology, Weifang People’s Hospital, Weifang, Shandong, China; ^3^ The Third Department of Geriatrics, Weifang People’s Hospital, Weifang, Shandong, China; ^4^ Hospital Preparation Center, Weifang People’s Hospital, Weifang, Shandong, China

**Keywords:** gastrointestinal cancers, CAR T cell therapy, engineered bacteria, tumor microenvironment, immunotherapy

## Abstract

The high incidence and mortality rates associated with gastrointestinal cancers represent a significant global health challenge. In recent years, CAR T cell therapy has emerged as a promising immunotherapeutic approach, demonstrating favorable clinical outcomes. However, the application of traditional CAR T cell therapy in gastrointestinal cancers faces numerous challenges, including the suppressive tumor microenvironment and limitations in anti-tumor efficacy. The application of engineered bacteria offers a novel strategy to enhance CAR T cell therapy by modulating the tumor microenvironment and boosting immune responses, potentially leading to improved therapeutic outcomes. This review synthesizes the current research advancements related to engineered bacteria-assisted CAR T cell therapy in gastrointestinal cancers, exploring its underlying mechanisms, clinical applications, and future developmental directions.

## 1 Introduction

Gastrointestinal (GI) cancers, which encompass malignancies of the digestive system, including colorectal, gastric, and esophageal cancers, represent a significant global health concern. With increasing incidence rates, these cancers are among the leading causes of cancer-related mortality worldwide (1). The complex interplay of genetic, environmental, and lifestyle factors contributes to the development of GI cancers, necessitating innovative therapeutic strategies to improve patient outcomes (2). Among these strategies, chimeric antigen receptor T-cell (CAR T-cell) therapy has emerged as a promising approach, particularly in hematological malignancies. However, its application in solid tumors, including GI cancers, poses unique challenges due to the heterogeneous nature of these tumors and the immunosuppressive tumor microenvironment (3).

CAR T-cell therapy involves genetically modifying T-cells to express a receptor that targets specific tumor antigens, allowing for more precise tumor cell destruction. The development of CAR T-cell therapy has progressed significantly since the first FDA-approved product in 2017, which targeted CD19 in B-cell malignancies (4). However, the success of CAR T-cell therapy in solid tumors has been limited by factors such as inadequate tumor infiltration, off-target effects, and T-cell exhaustion. These limitations highlight the need for adjunctive strategies to enhance the efficacy of CAR T-cell therapy in treating GI cancers (5).

One such adjunctive strategy involves the use of engineered bacteria. These microbes can selectively target tumor sites, modulate the immune response, and deliver therapeutic agents directly to the tumor microenvironment. Engineered bacteria can be designed to express immunomodulatory factors or chemotherapeutic agents, thereby enhancing the anti-tumor effects of CAR T-cells (6–8). This approach addresses the challenges associated with CAR T-cell therapy and opens new avenues for treatment by harnessing the unique properties of bacteria to improve therapeutic efficacy and safety.

In this review, we will explore the epidemiological landscape of GI cancers, the principles and evolution of CAR T-cell therapy, and the potential of engineered bacteria as a complementary strategy to enhance CAR T-cell therapy in the treatment of gastrointestinal malignancies. By integrating these innovative approaches, we aim to provide insights into the future of cancer therapy and the potential for improved outcomes in patients with GI cancers.

## 2 Main

### 2.1 The composition and role of the tumor microenvironment in gastrointestinal cancers

The tumor microenvironment (TME) plays a critical role in the development, progression, and therapeutic GI cancers. It is composed of various cellular and non-cellular components, including cancer cells, stromal cells, immune cells, extracellular matrix (ECM), and signaling molecules. The interaction between these components creates a complex network that can either promote or inhibit tumor growth. One of the key players in the TME is cancer-associated fibroblasts (CAFs), which are known to secrete growth factors and cytokines that support tumor proliferation and metastasis (9). Additionally, immune cells such as tumor-associated macrophages (TAMs) and myeloid-derived suppressor cells (MDSCs) contribute to an immunosuppressive environment, allowing tumors to evade immune surveillance (10).

The composition of the TME is influenced by various factors, including the metabolic state of the tumor, the presence of hypoxia, and the dysregulation of immune responses. For instance, hypoxia within the TME can lead to the selection of more aggressive cancer cell phenotypes and promote angiogenesis, further complicating treatment strategies (11). Moreover, the gut microbiome has emerged as a significant factor influencing the TME, with dysbiosis potentially facilitating tumor initiation and progression through immune modulation and inflammation (12). The TME also poses challenges for therapeutic interventions, particularly in the context of immunotherapy. The presence of immunosuppressive cells and cytokines can hinder the efficacy of treatments like CAR-T cell therapy, which has shown promise in hematologic malignancies but faces significant obstacles in solid tumors, including GI cancers (13). In summary, the TME in gastrointestinal cancers is a dynamic and complex entity that significantly influences tumor behavior and treatment outcomes. Ongoing research is crucial to unravel the mechanisms governing TME interactions and to identify potential therapeutic targets that can improve patient responses to treatment. The integration of insights from molecular biology, immunology, and microbiome studies will be vital in advancing the field of GI cancer therapy (8).

### 2.2 Research progress on CAR T Cell therapy in gastrointestinal cancers

CAR T cell therapy has emerged as a groundbreaking approach in the field of cancer immunotherapy, particularly in treating hematological malignancies. While, its application in solid tumors, especially GI cancers, presents unique challenges. These challenges include the identification of suitable target antigens, the complex TME, and safety concerns related to off-target effects and cytokine release syndrome (13). Recent studies have focused on optimizing CAR T cell designs and exploring innovative strategies to enhance their efficacy against GI cancers, such as colorectal, gastric, and pancreatic cancers.

One significant advancement in CAR T cell therapy for GI cancers is the identification of tumor-specific antigens, such as guanylyl cyclase C (GUCY2C) and claudin 18.2 (CLDN18.2). GUCY2C has shown promise due to its restricted expression in normal tissues and consistent overexpression in colorectal cancer, making it an ideal target for CAR T cell therapy (14). Similarly, CLDN18.2 has been recognized as a critical target in gastric and pancreatic cancers, with ongoing clinical trials demonstrating the safety and efficacy of CLDN18.2-targeted CAR T cells (15). These studies highlight the potential for CAR T cells to selectively target malignant cells while sparing normal tissues, thereby reducing the risk of toxicity.

Moreover, the engineering of CAR T cells to enhance their persistence and functionality within the TME is a crucial area of research. Innovations such as “armored” CAR T cells, which are designed to resist immunosuppressive signals from the TME, have been developed to improve therapeutic outcomes (16). Additionally, the incorporation of dual-targeting strategies, where CAR T cells are engineered to recognize multiple antigens, may help overcome the issue of antigen heterogeneity commonly observed in solid tumors (17). These approaches aim to enhance the ability of CAR T cells to infiltrate tumors and exert their cytotoxic effects effectively.

Despite these advancements, the clinical application of CAR T cell therapy in GI cancers is still in its infancy. Most clinical trials have been in early phases, and while some have reported promising results, challenges such as T cell exhaustion and limited durability of responses remain (5). The exploration of combination therapies, such as integrating CAR T cell therapy with immune checkpoint inhibitors or other immunotherapeutic agents, is being actively investigated to enhance overall efficacy and patient outcomes (18). As research progresses, the hope is to refine CAR T cell therapy for GI cancers, ultimately leading to improved treatment options for patients facing these challenging malignancies.

### 2.3 Characteristics of engineered bacteria and their applications in tumor therapy

Engineered bacteria have emerged as a promising tool in cancer therapy due to their unique properties, including the ability to target tumor cells selectively, modulate the immune response, and deliver therapeutic agents effectively. These bacteria can be categorized based on their genetic modifications and functional capabilities. For instance, some engineered strains are designed to express therapeutic proteins, such as cytokines or tumor antigens, which can enhance immune responses against tumors (19). Others are modified to produce oncolytic agents or to increase their retention in the tumor microenvironment, thereby maximizing therapeutic efficacy (20). The versatility of engineered bacteria allows for the development of personalized cancer therapies that can adapt to the specific characteristics of individual tumors, making them a valuable asset in the fight against cancer.

#### 2.3.1 Functions of engineered bacteria

Engineered bacteria can be classified into several categories based on their design and intended therapeutic functions. One major classification is based on their pathogenicity; for example, non-pathogenic strains such as *Escherichia coli* are often utilized for their safety profile in clinical applications (21). Another classification considers the type of modifications made to enhance their therapeutic potential. Genetic engineering techniques, such as CRISPR-Cas systems, allow for precise alterations in bacterial genomes to enhance their functionality, including improved targeting of tumor cells and modulation of immune responses (22). Engineered bacteria can also be designed to produce various therapeutic agents, including cytokines, enzymes, or even nanoparticles, which can be delivered directly to the tumor site, thereby increasing the local concentration of the drug while minimizing systemic side effects (8, 19). And bacteria can be designed to mediat metabolic reprogramming like lactate depletion to potentiate antitumor immunity (23). The multifunctional capabilities of engineered bacteria position them as a versatile platform for innovative cancer therapies, capable of addressing the challenges posed by traditional treatment methods.

#### 2.3.2 Role of engineered bacteria in enhancing immune responses

Engineered bacteria play a crucial role in enhancing immune responses against tumors through various mechanisms. They can stimulate both innate and adaptive immunity, leading to a robust anti-tumor effect. For instance, certain engineered strains can activate dendritic cells and promote the proliferation of T cells, which are essential for effective immune surveillance and tumor eradication (24). Additionally, engineered bacteria can be designed to deliver immune-modulating agents, such as checkpoint inhibitors, directly into the tumor microenvironment, thereby overcoming the immunosuppressive barriers often present in tumors (25). The ability of these bacteria to selectively proliferate in hypoxic tumor regions further enhances their therapeutic potential, as they can compete for nutrients and space with tumor cells while simultaneously releasing therapeutic agents (20). Unlike the nanoparticle systems rely on passive EPR-driven diffusion, often failing to reach hypoxic tumor cores, bacteria enable dynamic, self-replicating therapy, nanoparticles face dose limitations and poor penetration (24, 25). The integration of engineered bacteria into cancer immunotherapy represents a significant advancement in the development of more effective and personalized treatment strategies.

#### 2.3.3 Comprehensive summary of bacterial strains in tumor therapy

Engineered bacterial strains demonstrate distinct mechanisms and therapeutic potential in tumor therapy. *Salmonella typhimurium* VNP20009 requires slyA, STM3120, and htrA genes (26) for tumor colonization and immune activation via TNF-α/IL-1β induction (27), though its clinical translation has been limited by attenuated tumor colonization in humans (28). In contrast, S.t ΔppGpp, engineered with cytolysin A (ClyA) and radiotherapy synergy, enables dual tumor lysis and bioluminescence imaging (29). The *Salmonella enterica* A1-R strain directly lyses tumors through matrix metalloproteinase secretion, showing broad efficacy in multiple tumor models (30), while the LT2-derived CRC2631 strain balances safety and tumor-selective replication (31). The obligate anaerobic *Salmonella* eliminates systemic toxicity by hypoxia-targeted gene regulation, achieving robust efficacy in breast cancer models (32). Despite these advances, challenges persist, including host immune clearance, strain optimization for motility, and reconciling attenuation with therapeutic potency (27). Future directions emphasize metabolic engineering *E. coli* and synthetic biology to design tumor-localized strains like YB1 (32), aiming to synergize bacterial therapies with checkpoint inhibitors and chemotherapy.

### 2.4 Mechanisms of Engineered Bacteria-assisted CAR T Cell therapy

**FIGURE 1:**
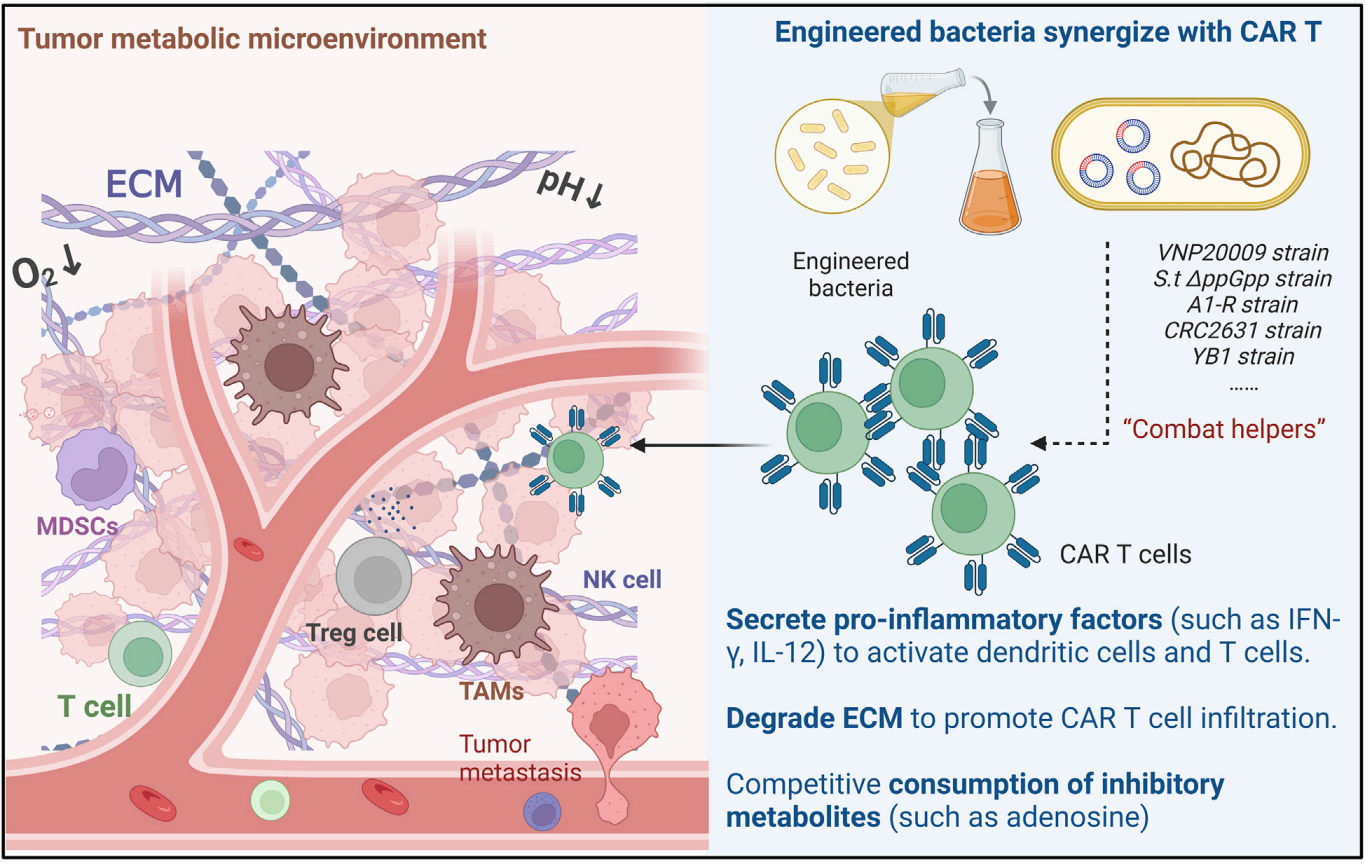
Mechanisms of Engineered Bacteria-Assisted CAR T Cell Therapy: Engineered bacteria can assist CAR-T cells through three main ways: Cytokine secretion, Reshaping ECM and Metabolic remodeling.

The integration of engineered bacteria into CAR T cell therapy represents a novel approach aimed at enhancing the efficacy of cancer treatments (Figure 1). This strategy leverages the unique properties of bacteria to modulate the TME and improve the therapeutic outcomes of CAR T cells. Engineered bacteria can be designed to deliver therapeutic agents, such as cytokines, directly to tumor sites, thereby creating a more favorable environment for CAR T cell activity. For instance, non-pathogenic strains like *E. coli* have been utilized to express immune-activating cytokines, which can stimulate immune responses and facilitate the infiltration of T cells into tumors. This dual action not only enhances the local immune response but also helps overcome some of the immunosuppressive barriers typically present in solid tumors, thereby improving the overall effectiveness of CAR T cell therapies (21).

#### 2.4.1 Enhancing CAR T Cell activity by modulating the tumor microenvironment

The tumor microenvironment plays a critical role in determining the success of CAR T cell therapies. Engineered bacteria can significantly alter the TME to enhance CAR T cell activity. For example, bacteria can be engineered to produce specific cytokines that counteract the immunosuppressive signals present in the TME. One study demonstrated that non-pathogenic *E. coli* expressing a decoy-resistant IL-18 mutein could activate natural killer (NK) cells and enhance their trafficking into tumors, ultimately leading to improved survival in treatment-resistant cancer models (21). Furthermore, the use of nanoengineered CAR T cells has shown promise in remodeling the TME through photothermal strategies, like INPs engineered CAR-T biohybrids (CT-INPs) not only retain the original activities and functions of CAR-T cells, but it is further armed with fluorescent tracing and microenvironment remodeling abilities (33). And TGF-β blocking together with photothermal therapy promote tumor-targeted migration and long-term antitumor activity of CAR-T, which disrupt the cellsextracellular matrix and promote better infiltration of CAR T cells into solid tumors (34). Additionally, other approaches have involved the use of inverted cytokine receptors to convert inhibitory signals into stimulatory ones, thereby enhancing CAR T cell function in hostile environments like pancreatic tumors (35). These strategies underscore the potential of engineered bacteria to create a more conducive environment for CAR T cell action, thereby improving therapeutic outcomes. One preclinical study showed the ability of *Salmonella* + Alb-IL2 to serve as a novel therapeutic approach to induce T cell-mediated antitumor immunity in a murine model of cancer (36). Vincent et al. engineered bacteria that colonize solid tumours to deliver synthetic antigens and generated CAR-T cells specific for these antigens. This approch effectively eliminated tumours in mouse models of breast and colon cancer (7).

#### 2.4.2 Promoting immune cell infiltration and activation

In addition to modifying the TME, engineered bacteria can facilitate the infiltration and activation of various immune cells, thereby enhancing the overall immune response against tumors. The presence of bacteria in the TME can attract immune cells, such as T cells and NK cells, to the tumor site. This recruitment is crucial for mounting an effective anti-tumor response. For instance, the engineering of CAR T cells to express chemokines like CXCL9 has been shown to improve T cell trafficking to tumors, resulting in enhanced antitumor efficacy (37). Moreover, the use of oncolytic viruses combined with CAR T cells has demonstrated the ability to increase immune cell infiltration (38), though new concerns like the oncolytic virus-derived type I interferon restrict CAR T cell therapy effect persist (39). Oncolytic mineralized bacteria can lead to the activation of myeloid cells within the tumor, which further supports the development of a robust antitumor immune response (40). Engineered immune cells and bacteria classically activated macrophages within tumor tissue, which directly kill tumor cells (41). Overall, the strategic use of engineered bacteria not only enhances CAR T cell activity but also promotes a more comprehensive immune response, thereby improving the potential for successful cancer immunotherapy.

### 2.5 Future development directions and challenges

#### 2.5.1 Future directions in combination therapies

The integration of engineered bacteria with CAR T cell therapy represents a promising frontier in cancer treatment. CAR T cell therapy has revolutionized the management of certain hematologic malignancies, but its application in solid tumors has been limited due to the immunosuppressive tumor microenvironment and the challenges of T cell infiltration. Engineered bacteria, with their unique ability to selectively target tumors and modulate the immune response, offer a novel strategy to enhance the efficacy of CAR T cell therapies. Research has shown that bacteria can be engineered to produce therapeutic agents or stimulate immune pathways that can synergize with CAR T cells, potentially overcoming the barriers faced in solid tumor environments (42).

To optimize the synergy between engineered bacteria and CAR T-cell therapy, it is crucial to precisely modulate the TME. This can be achieved by designing engineered bacteria that secrete specific cytokines (such as IL-12 or IL-18 mutants) or metabolites to directly alter the immunosuppressive TME, enhancing CAR T-cell infiltration and activity (10, 43). Furthermore, developing bacteria that respond to TME signals like hypoxia or pH changes can enable controlled, on-demand release of therapeutic factors, minimizing systemic toxicity (44, 45). A dual-target strategy, combining tumor-specific antigens and chemokine receptors, would improve the targeting of gastrointestinal tumors while reducing off-target effects (45). Additionally, utilizing CRISPR-Cas or phage integration technologies to engineer non-pathogenic bacteria with suicide switches ensures the safe elimination of bacteria after treatment (22).

In terms of treatment innovation, combining engineered bacteria with immune checkpoint inhibitors, such as PD-1/PD-L1 antagonists, can help overcome T-cell exhaustion and improve CAR T-cell efficacy (46). Integrating oncolytic viruses or nanotechnology into bacterial delivery systems, including hybrid bacterial-virus carriers, could further enhance tumor disruption and facilitate CAR T-cell infiltration (47). Personalized therapies tailored to a patient’s unique microbiota could improve treatment tolerance, while multi-antigen CAR T-cell therapies, augmented by engineered bacteria, would address tumor heterogeneity (48).

#### 2.5.2 Challenges and potential strategies

**FIGURE 2:**
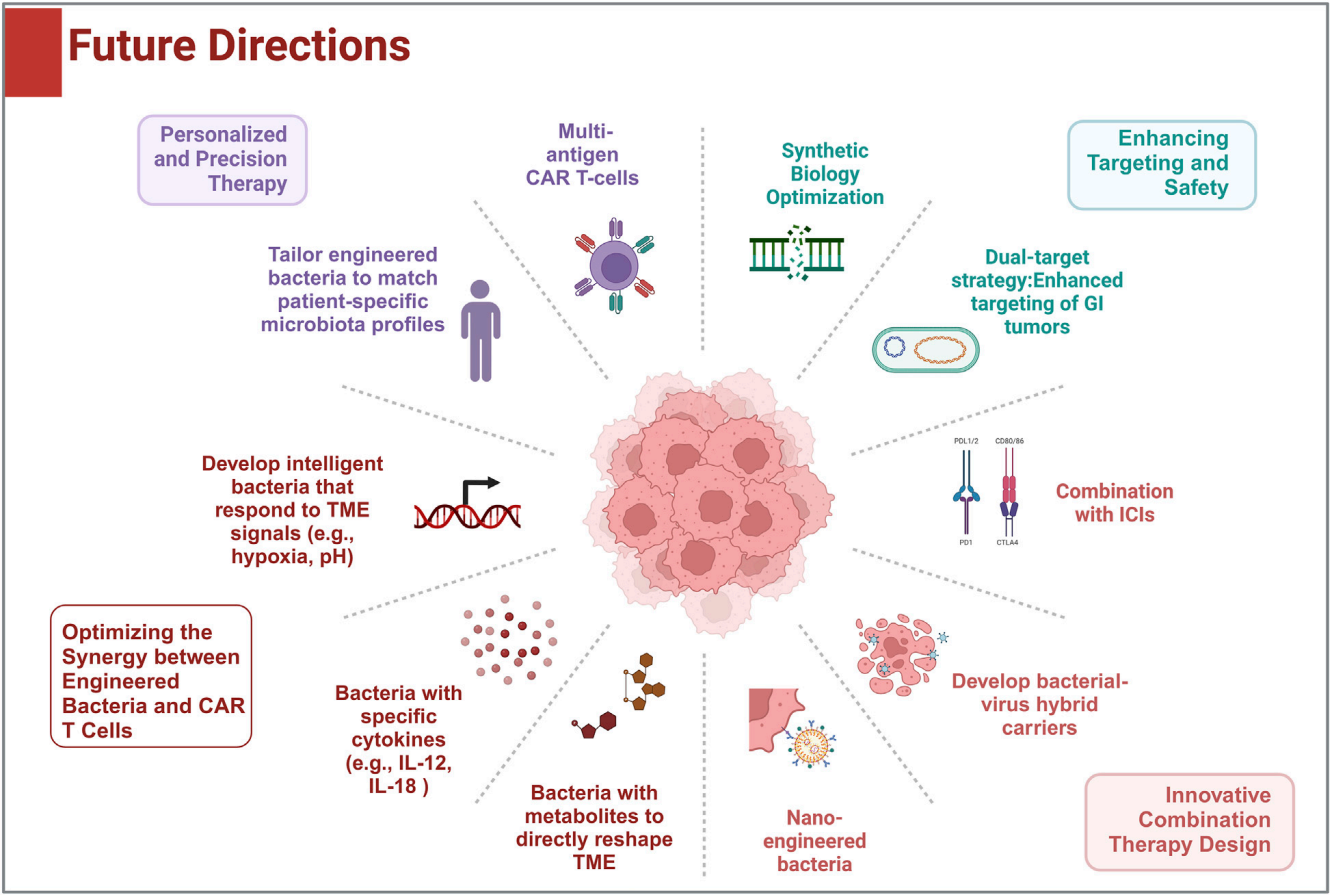
Future development directions: This figure shows the potential research and clinical directions for the combination of CAR-T therapies with engineered bacteria in solid tumor treatment.

We also addresses safety risks in bacterial-mediated tumor therapy, focusing on bacterial toxicity and immune hyperactivation. Live bacterial therapy carries certain risks, including infection risk and immunogenicity. We propose engineered inducible kill switches (e.g., toxin-antitoxin systems controlled by tumor-specific promoters) to restrict bacterial proliferation post-treatment (49) (Figure 2). Moreover, bacterial components (e.g., LPS) may trigger cytokine storms *via* cGAS-STING/NF-κB pathways (50). *In vitro in vivo* trials are necessary to test these potential side effects. And dual-control circuits (oxygen/pH-responsive elements) may ensure bacterial clearance upon immune overactivation, balancing efficacy and safety in the future. To ensure clinical applicability, standardized production methods for both engineered bacteria and CAR T-cells must be developed for large-scale use. Early clinical trials, particularly in cancers like colorectal or gastric cancer, can validate the safety and efficacy of these combined therapies. Finally, long-term safety and resistance monitoring are critical, requiring strategies to manage potential inflammation and tumor escape mechanisms.

## 3 Conclusion

The integration of engineered bacteria to assist CAR T-cell therapy presents a promising frontier in the treatment of gastrointestinal cancers. This innovative approach harnesses the unique properties of bacteria to enhance the efficacy of CAR T-cell therapies, potentially overcoming many of the limitations currently faced in the field. The ability of engineered bacteria to localize to tumor sites, modulate the tumor microenvironment, and activate immune responses could lead to improved therapeutic outcomes for patients suffering from these challenging malignancies. However, this promising strategy is not without its challenges. Key issues that require further exploration include the optimization of bacterial delivery systems, the assessment of safety profiles, and the understanding of the complex interactions between engineered bacteria, CAR T-cells, and the host immune system. Moreover, the heterogeneity of gastrointestinal tumors adds another layer of complexity, necessitating tailored approaches to maximize therapeutic effectiveness.

In balancing the various research perspectives and findings, it is crucial to adopt a multidisciplinary approach that incorporates insights from immunology, microbiology, and oncology. Collaboration among researchers, clinicians, and industry stakeholders will accelerate the development of this innovative therapy. Future research directions should prioritize the design of clinical trials that evaluate not only the efficacy but also the safety and tolerability of this combined therapy. Additionally, investigating the mechanisms by which engineered bacteria enhance CAR T-cell function will be vital for optimizing treatment strategies. As we look ahead, the potential for engineered bacteria to revolutionize CAR T-cell therapy in gastrointestinal cancers is significant. By addressing the critical questions and challenges identified in this review, the field can move toward a deeper understanding of how to effectively leverage this synergy.
